# Antibody Avidity and Neutralizing Response against SARS-CoV-2 Omicron Variant after Infection or Vaccination

**DOI:** 10.1155/2022/4813199

**Published:** 2022-08-31

**Authors:** Francesca Dapporto, Serena Marchi, Margherita Leonardi, Pietro Piu, Piero Lovreglio, Nicola Decaro, Nicola Buonvino, Angela Stufano, Eleonora Lorusso, Emilio Bombardieri, Antonella Ruello, Simonetta Viviani, Eleonora Molesti, Claudia Maria Trombetta, Alessandro Manenti, Emanuele Montomoli

**Affiliations:** ^1^VisMederi srl, Siena, Italy; ^2^Departmente of Molecular and Developmental Medicine, University of Siena, Siena, Italy; ^3^Vismederi Research Srl, Siena, Italy; ^4^Interdisciplinary Department of Medicine, Section of Occupational Medicine, University of Bari, Bari, Italy; ^5^Department of Veterinary Medicine, University of Bari, Bari, Italy; ^6^U.O.C. Penitentiary Medicine-Department of Territorial Care, Bari Local Health Authority, Bari, Italy; ^7^Humanitas Gavazzeni, Bergamo, Italy

## Abstract

**Background:**

The recently emerged SARS-CoV-2 Omicron variant exhibits several mutations on the spike protein, enabling it to escape the immunity elicited by natural infection or vaccines. Avidity is the strength of binding between an antibody and its specific epitope. The SARS-CoV-2 spike protein binds to its cellular receptor with high affinity and is the primary target of neutralizing antibodies. Therefore, protective antibodies should show high avidity. This study aimed at investigating the avidity of receptor-binding domain (RBD) binding antibodies and their neutralizing activity against the Omicron variant in SARS-CoV-2 infected patients and vaccinees.

**Methods:**

Samples were collected from 42 SARS-CoV-2 infected patients during the first pandemic wave, 50 subjects who received 2 doses of mRNA vaccine before the Omicron wave, 44 subjects who received 3 doses of mRNA vaccine, and 35 subjects who received heterologous vaccination (2 doses of adenovirus-based vaccine plus mRNA vaccine) during the Omicron wave. Samples were tested for the avidity of RBD-binding IgG and neutralizing antibodies against the wild-type SARS-CoV-2 virus and the Omicron variant.

**Results:**

In patients, RBD-binding IgG titers against the wild-type virus increased with time, but remained low. High neutralizing titers against the wild-type virus were not matched by high avidity or neutralizing activity against the Omicron variant. Vaccinees showed higher avidity than patients. Two vaccine doses elicited the production of neutralizing antibodies, but low avidity for the wild-type virus; antibody levels against the Omicron variant were even lower. Conversely, 3 doses of vaccine elicited high avidity and high neutralizing antibodies against both the wild-type virus and the Omicron variant.

**Conclusions:**

Repeated vaccination increases antibody avidity against the spike protein of the Omicron variant, suggesting that antibodies with high avidity and high neutralizing potential increase cross-protection against variants that carry several mutations on the RBD.

## 1. Introduction

Since the first isolation of SARS-CoV-2 in January 2020 in China [1], several viral variants have been detected. The Omicron BA.1 (Pango lineage B.1.1.529) variant was first reported in South Africa and Botswana in November 2021; since then, it has spread worldwide [2] and was included among “variants of concern” (VoCs) [3]. Omicron is the most divergent variant and is characterized by more than 50 mutations, 30 of which on the spike (S) protein. Notably, 15 mutations are located in the receptor-binding domain (RBD) of the S protein, and some of them are shared with other variants [4–6].

The S protein plays an essential role in SARS-CoV-2 infection and constitutes the main target of neutralizing antibodies [7]. The current vaccine formulations are designed to target the S protein of the wild-type (wt) virus, derived from the original Wuhan strain, and have proved to offer a high degree of protection. Currently, five vaccines have been authorized in Europe [8]: The BNT162b2 (Pfizer-BioNTech) and mRNA-1273 (Moderna) vaccines were developed by using the mRNA vaccine platform, Ad26.COV2.S (Janssen/Johnson & Johnson) and ChAdOx1-S (AstraZeneca) are adenovirus vectored vaccines, and NVX-CoV2373 (Novavax) is a recombinant SARS-CoV-2 nanoparticle vaccine.

Antibody binding to an antigen is a noncovalent interaction [9], and it has been shown that the affinity of antibodies can increase over time, through the affinity maturation process. This is a consequence of somatic hypermutation occurring in the germinal centers, thus generating antibodies that bind more strongly to the antigen [10]. The strength of binding between immunoglobulin (Ig) and its specific target epitope is defined as avidity [11].

Antibodies induced by viral infections, or by vaccination with live-attenuated viruses, can persist for decades. However, most vaccine formulations based on protein antigens require repeated vaccinations in order to generate immunological memory and to maintain antibody responses above protective levels [12]. The level of antigen–antibody binding avidity, a qualitative response index, can also correlate with protection and can potentially be enhanced by repeated immunization. Conversely, inadequate levels of avidity maturation can heighten susceptibility to viral infection [13].

Immune responses towards the SARS-CoV-2 nucleoprotein, S protein, and RBD following natural infection are characterized by incomplete avidity maturation, as also observed in other coronavirus infections [14, 15]. By contrast, studies conducted on recipients of one or two doses of vaccines have reported an increase in antibody avidity, suggesting potential antibody maturation after vaccination [16, 17].

To evaluate the potential of the avidity index (AI) as a marker of protection against RBD-mutated variants, we investigated the avidity of RBD-binding antibodies and their neutralizing activity against the wt SARS-CoV-2 virus and the Omicron variant in SARS-CoV-2 infected patients and subjects who received homologous or heterologous vaccinations.

We found that vaccinated subjects show higher avidity than patients. Moreover, subjects who received 3 doses of vaccine reach high IgG avidity and neutralizing activity towards Omicron variant.

## 2. Materials and Methods

### 2.1. Study Population

A total of 176 serum samples were collected from 42 SARS-CoV-2 infected patients hospitalized at Humanitas Gavazzeni (Bergamo, Italy) during the first pandemic wave (March-May 2020). Patient characteristics and study procedures are described elsewhere [18]. Samples were collected at different time-points (on hospital admission, day 2, day 6, days 12–14, days 18–20, days 27–30, and discharge/decease). This study was approved by the Ethics Committee of the University of Siena (approval number 17373,) and by the Ethics Committee of Humanitas Gavazzeni (approval number 236). All serum samples have been fully anonymized before testing.

Fifty (50) and forty-four (44) serum samples were collected from inmates of the Bari correctional facility (Apulia, Italy) who had been vaccinated with one of the two mRNA vaccines approved in Italy (mRNA-1273 and BNT162b2). Samples were collected 21 days (mean) after the 2nd and 3rd doses.

Thirty-five (35) serum samples were collected from employees of the University of Bari 42 days (mean) after vaccination with a booster dose (3rd dose) of one of the two available mRNA vaccines (mRNA-1273 and BNT162b2). These subjects had initially received 2 doses of the adenovirus-based vaccine ChAdOx1-S (AstraZeneca).

Samples from subjects who received 2 doses of vaccine were collected before the Omicron wave (May-June 2021), while samples from subjects who received 3 doses of vaccine were collected during the Omicron wave in January 2022.

All subjects provided informed consent to participate in the study and data processing prior to the start of the study and after receiving a briefing on the study by medical personnel. The research protocol was approved by the Ethics Committee of the University Hospital of Bari (n. 6955, prot. N. 0067544–02082021).

### 2.2. Cell Lines and Viruses

Vero E6 cells (American Type Colture Collection [ATCC] #CRL-1586/Vero C1008) were grown in high-glucose Dulbecco's Modified Eagle's Medium (DMEM) (Euroclone, Pero, Milan) supplementend with 2 mM L-Glutamine (Euroclone, Pero, Milan), 100 U/mL of penicillin-100 *μ*g/mL streptomycin (P/S Gibco, Life Technologies) ,and 10% Foetal Bovine Serum (FBS) (complete DMEM) (Euroclone, Pero, Milan). Cells were maintained at 37°C in a humified 5% CO_2_ atmosphere. 18-24 hours before execution of the microneutralization (MN) assay, 96-well plates were seeded with 100 *μ*L/well of Vero E6 cell suspension (1.5 × 10^5^ cell/mL) diluted in complete DMEM, supplemented with 2% FBS, and incubated at 37°C with 5%CO_2_ until use.

SARS-CoV-2 (2019-nCov/Italy-INMI1 strain) wt virus was purchased from the European Virus Archive goes Global (EVAg, Spallanzani Institute, Rome). The Omicron variant was kindly provided by Prof. Piet Maes, NRC UZ/KU Leuven (Leuven, Belgium). The Omicron sequence is registered on the GISAID portal with the following ID: EPI_ISL_6794907.

Viral propagation was performed in 175cm^2^ tissue-culture flasks pre-seeded with 50 mL of Vero E6 cells (1 × 10^6^ cells/mL) diluted in DMEM 10%FBS. After 18-20-hour incubation at 37 °C, 5%CO_2_, flasks were washed twice with sterile Dulbecco's phosphate buffered saline (DPBS) and inoculated with the SARS-CoV-2 virus at a multiplicity of infection (MOI) of 0.001. The sub-confluent cell monolayer was incubated with the virus for 1 hour at 37 °C, 5%CO_2_; the flasks were filled with 50 mL of DMEM 2%FBS and incubated at 37 °C, 5%CO_2_. Cells were checked daily until an 80-90% cytopathic effect (CPE) was observed. Supernatants of the infected cultures were harvested, centrifuged at 469×g for 5 minutes at 4 °C to remove cell debris, and stored at − 80 °C.

The propagated viral stocks were titrated in 96-well plates previously seeded with Vero E6 cells. Ten-fold serial dilutions of virus (10^−1^ to 10^−11^) were incubated with cells and checked for CPE for a total of 72 hours (wt virus) or 96 hours (Omicron variant). The viral titer was calculated by using the 50% tissue culture infectious dose per mL (TCID50/mL) as the endpoint and was defined as the reciprocal of the highest virus dilution yielding at least 50% CPE in the infected wells, according to the Reed and Muench formula [19].

### 2.3. In-House Enzyme-Linked Immunosorbent Assay (ELISA)

IgG determination in serum samples was performed by an in-house ELISA RBD [20]; 96-well ELISA plates were coated with 1 *μ*g/mL of purified recombinant Wuhan SARS-CoV-2 Spike-RBD protein (Arg319-Phe541) (Sino Biological) expressed and purified from HEK-293 cells. Plates were incubated at 4 °C overnight and washed three times with 300 *μ*L/well of tris buffered saline (TBS)-0.05% Tween20 (T-TBS) and blocked for 1 hour at 37 °C with a solution of T-TBS containing 5% of non-fat dry milk (NFDM, Euroclone, Pero, Italy). Samples were two-fold serially diluted in 5% NFDM/T-TBS. After washing steps, 100 *μ*L of each serial dilution was added to plates, which were incubated for 1 hour at 37 °C. Subsequently, plates were washed and 100 *μ*L of Goat anti-Human IgG-Fc Horse Radish Peroxidase (HRP)-conjugated antibody (Bethyl Laboratories, Montgomery, USA) diluted 1 : 100,000 in 5% NFDM/T-TBS was added to each well. Plates were incubated at 37 °C for 30 minutes and, after washing steps and the addition of 100 *μ*L/well of 3,3′,5,5′-tetramethylbenzidine (TMB) substrate (Bethyl Laboratories, Montgomery, USA), were incubated in the dark at room temperature for 20 minutes. The reaction was stopped by adding 100 *μ*L of 0.5 M hydrochloric acid solution (Fisher Chemical, Milan, Italy) and read within 20 minutes at 450 nm with a SpectraMax ELISA plate (Medical Device) reader. A cut-off value was defined as 3 times the average of optical density (OD) values from blank wells (background: no addition of analyte). Samples with ODs below the cut-off value on first dilution were classified as negative, while samples with ODs at the lowest dilution above the cut-off value were classified as positive [21].

### 2.4. IgG Avidity ELISA

The IgG avidity ELISA was performed as previously reported [22]. Briefly, serum samples were standardized to a dilution that yielded an OD of 1 ± 0.3 in ELISA, and after 1 hour of samples incubation, 1.5 M sodium thiocyanate (NaSCN) was added to samples and incubated for 1 hour. The test was continued as in the previously described ELISA.

The AI was calculated as the percentage of IgG detected after treatment with the NaSCN agent, after subtracting the blank value from each OD: (Average OD of sample treated with 1.5 M NaSCN/Average OD of untreated sample) × 100.

AIs below 30% were deemed to indicate low avidity: from 31% to 50%, intermediate avidity; and above 50%, high avidity [23].

### 2.5. CPE-Based Microneutralization Assay

Ten 2-fold serial dilutions of the serum samples (starting dilution 1 : 10) were prepared in duplicate in complete DMEM 2%FBS in 96-well plates. Plates were incubated for 1 hour at 37 °C with a standard concentration of virus (sample:virus ratio 1 : 1) [24]. Following incubation, the virus-sample mixture was added to sub-confluent Vero E6 cells. After 72 hours (wt virus) or 96 hours (Omicron variant), cells were inspected for the presence of CPE. The highest sample dilution able to completely inhibit viral growth was regarded as the neutralization titer.

### 2.6. Statistical Analysis

The results were evaluated for normal distribution by D'Agostino and Pearson, Shapiro-Wilk, and Kolmogorov-Smirnov normality tests. Statistically significant differences between antibody titers and AIs were determined by Kruskal-Wallis and Dunn's multiple comparisons test. The AIs and neutralizing antibody titers were normalized with respect to their minimum values evaluated for wt data. In addition, the normalized neutralizing antibody titers underwent a log-transformation (base 2). The relationship between the neutralizing antibody titer and avidity in the vaccinated cohorts was assessed by a multiple regression model that also considered the interaction with the virus strains and the number of vaccine doses. *p* values < 0.05 were considered statistically significant. Statistical analyses were performed and graphs constructed by GraphPad Prism v. 9.0 (GraphPad Software, San Diego, USA) and R (version 4.0.3).

## 3. Results

### 3.1. Time Course of RBD-Binding IgG Titers, AIs, and Neutralizing Antibody Titers in SARS-CoV-2 Infected Patients

**Figure 1:**
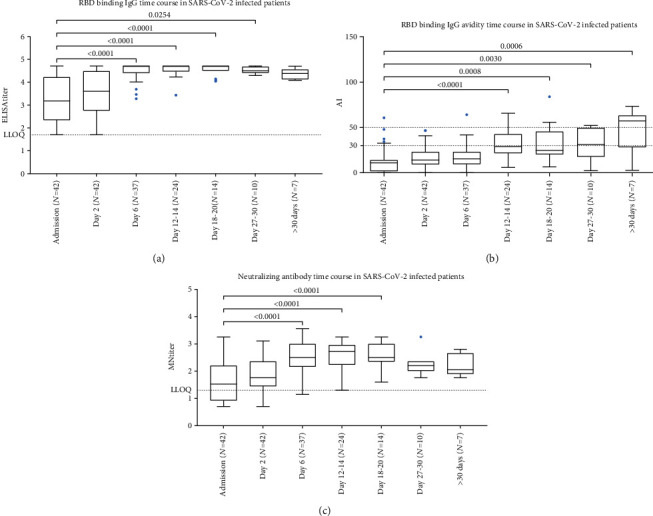
RBD binding IgG titers (a), RBD binding IgG antibody avidity (b), and neutralizing antibody titers (c) to SARS-CoV-2 wt virus in SARS-CoV-2 infected patients by time after hospital admission. RBD binding IgG titers which exceeded the last dilution (>51200) were plotted as 51200 titers. The antibody avidity was expressed as avidity index (AI). Tukey boxplots show outlier values (dots), medians (middle line), and third and first quartiles (boxes), while the whiskers display the minimum and maximum values. Horizontal dashed line represents the lower limit of quantification (LLOQ) of ELISA and microneutralization (MN) assay and AI range (low, intermediate, and high). Statistically significant differences were analyzed by Kruskal-Wallis and Dunn's multiple comparisons test (*p* < 0.05).

Samples collected from SARS-CoV-2 infected patients during their hospital stay were tested for RBD-binding IgG, antibody avidity, and neutralizing antibody (Figure 1).

Both RBD-binding IgG and neutralizing antibody titers temporally increased during hospitalization and peaked from day 6 to day 18-20 (*p* < 0.0001 vs hospital admission for both antibodies) before beginning to plateau or decrease (Figures 1(a) and 1(c)). AIs showed a significant increase (*p* < 0.0001) (Figure 1(b)). Specifically, the median AI on hospital admission was 10.8% (range 0.0-60.6) and significantly increased from day 12-14 (28.75%, range 5.9-65.6; *p* < 0.0001 vs hospital admission), reaching 57.2% (range 2.7-73.4) 30 days or more after admission. Low, intermediate, and high AIs were recorded in 47.6%, 33.3%, and 19.1% of patients, respectively, during the entire hospital stay.

### 3.2. RBD-Binding IgG Titers, AIs, and Neutralizing Antibody Titers in Vaccinated Subjects

**Figure 2:**
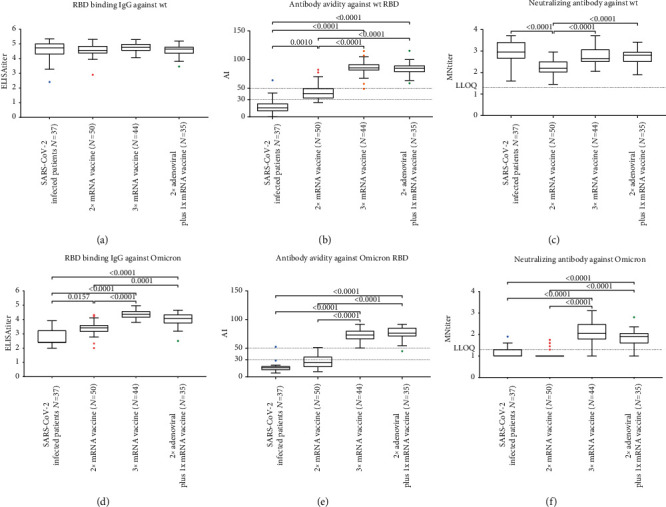
RBD binding IgG antibody avidity and neutralizing antibody titers to SARS-CoV-2 in SARS-CoV-2 infected patients and vaccinated cohorts: (a) RBD binding IgG antibody titers against wt virus; (b) RBD binding IgG antibody avidity against wt virus; (c) neutralizing antibody titers against wt virus; (d) RBD binding IgG antibody titers against Omicron variant; (e) anti-RBD IgG antibody avidity against Omicron variant; and (f) neutralizing antibody titers against Omicron variant. The antibody avidity was expressed as avidity index (AI). Tukey boxplots show outlier values (dots), medians (middle line), and third and first quartiles (boxes), while the whiskers display the minimum and maximum values. Horizontal dashed line represents the lower limit of quantification (LLOQ) of microneutralization (MN) assay and AI range (low, intermediate, and high). Statistically significant differences were analyzed by Kruskal-Wallis and Dunn's multiple comparisons test (*p* < 0.05).

Samples collected from subjects who had received 2 doses of mRNA vaccine, 3 doses of mRNA vaccine, or 2 doses of adenovirus-based vaccine and a booster dose of mRNA vaccine were tested for RBD-binding IgG, antibody avidity, and neutralizing antibody (Figures 2(a)–2(c)).

RBD-binding IgG titers, no differences were observed among the three cohorts, while neutralizing antibody titers were significantly higher in subjects who had received 3 doses of vaccine than in those who had received 2 doses (*p* < 0.0001 both for 3 doses of mRNA vaccine and for 2 doses of adenoviral vaccine plus 1 dose of mRNA).

The median AIs were 40.6% (range 25.1-82.3), 85.5% (range 49.0-114.8), and 85.0% (range 58.7-115.4) in subjects who had received 2 doses of mRNA vaccine, 3 doses of mRNA vaccine, and 2 doses of adenovirus-based vaccine plus a booster dose of mRNA vaccine, respectively. A significantly higher AI was observed in subjects who had received 3 doses of vaccine rather than 2 (*p* < 0.0001 both for 3 doses of mRNA vaccine and for 2 doses of adenovirus-based vaccine plus a booster dose of mRNA vaccine), while no differences were found between subjects who had received 3 doses of mRNA vaccine and those who had received 2 doses of adenoviral vaccine plus 1 dose of mRNA. High AIs were found in 22.0%, 97.7%, and 100.0% of subjects who had received 2 doses of mRNA vaccine, 3 doses of mRNA vaccine, and 2 doses of adenoviral plus 1 dose of mRNA vaccine, respectively.

### 3.3. Comparison of AIs and Neutralizing Antibody Titers between SARS-CoV-2 Infected Patients and Vaccinated Subjects

RBD-binding IgG titers, AIs, and neutralizing antibody titers against the wt virus were compared between SARS-CoV-2 infected patients and vaccinated subjects (Figures 2(a)–2(c)). In this comparison, only samples collected from patients on day 6 after hospitalization were selected, since these showed the highest neutralizing antibody titers against the wt virus [18].

Neutralizing antibody titers against wt observed in patients were similar to those observed in subjects who had received 3 doses of mRNA vaccine or 2 doses of adenovirus-based vaccine plus a booster dose of mRNA vaccine and significantly higher than in subjects who had received 2 doses of mRNA vaccine (*p* < 0.001) (Figure 2(c)).

No significant differences in RBD-binding IgG titers were observed among all the study cohorts (*p* = 0.172) (Figure 2(a)), while subjects who had received at least 2 doses of vaccine showed significantly higher AIs than patients (*p* = 0.001 vs 2 doses of vaccine and *p* < 0.0001 vs 3 doses of mRNA vaccine or 2 doses of adenoviral vaccine plus 1 dose of mRNA vaccine) (Figure 2(b)).

### 3.4. Comparison of AIs and Neutralizing Antibody Titers against the Omicron Variant in SARS-CoV-2 Infected Patients and Vaccinated Subjects

RBD-binding IgG titers, AIs, and neutralizing antibody titers against the Omicron variant were compared between SARS-CoV-2 infected patients and vaccinated subjects (Figures 2(d)–2(f)).

Neutralizing antibody titers against the Omicron variant were lower in patients than in subjects who had received 3 doses of vaccine (*p* < 0.0001), but similar to those observed in subjects who had received 2 vaccine doses (Figure 2(f)). Patients also showed significantly lower RBD-binding IgG titers than the other cohorts (*p* = 0.0157 vs 2 doses of mRNA vaccine and *p* < 0.0001 vs 3 doses of vaccine) (Figure 2(d)). With regard to AIs, similar patterns emerged; in patients, the values were similar to those seen in subjects who had received 2 doses of mRNA vaccine and significantly lower than in subjects who had received 3 doses of vaccine (*p* < 0.0001) (Figure 2(e)).

### 3.5. Relationship between Avidity and Neutralizing Activity Following Vaccination

Since no differences were observed between subjects who had received 3 doses of mRNA vaccine and those who had received 2 doses of adenovirus-based vaccine plus a booster dose of mRNA vaccine, we conducted a multiple regression analysis in order to determine whether antibody avidity and the number of vaccine doses (“2 doses” as reference, and “3 doses”) and virus strain (“wt”, and “Omicron” as reference) could predict the MN results.

First, we determined whether RBD-binding antibody titers were associated with neutralizing antibody. A significant association was found between RBD-binding IgG titers and neutralizing antibody titers (Figure 1S(a) and 1S(b)) for both the wt virus (slope = 0.52, *p* < 0.0001, *r* = 0.44, and *N* = 128) and Omicron variant (slope = 0.76, *p* < 0.0001, *r* = 0.74, and *N* = 128), without considering the number of vaccine doses received. When the number of vaccine doses was included in the model (Figure 1S(c) and 1S(d)), a significant association between RBD-binding IgG titers and neutralizing titers was found for wt virus for both subjects who received 2 (slope = 0.29, *p* = 0.1, *r* = 0.36, and *N* = 49) or 3 doses of vaccine (slope = 0.62, *p* < 0.0001, *r* = 0.58, and *N* = 79). However, a significant association between RBD-binding IgG titres and neutralizing titers for Omicron variant was found only in subjects who received 3 doses of vaccine (slope = 0.61, *p* < 0.0001, *r* = 0.57, and *N* = 79), but not in subjects who received 2 doses of vaccine.

**Figure 3:**
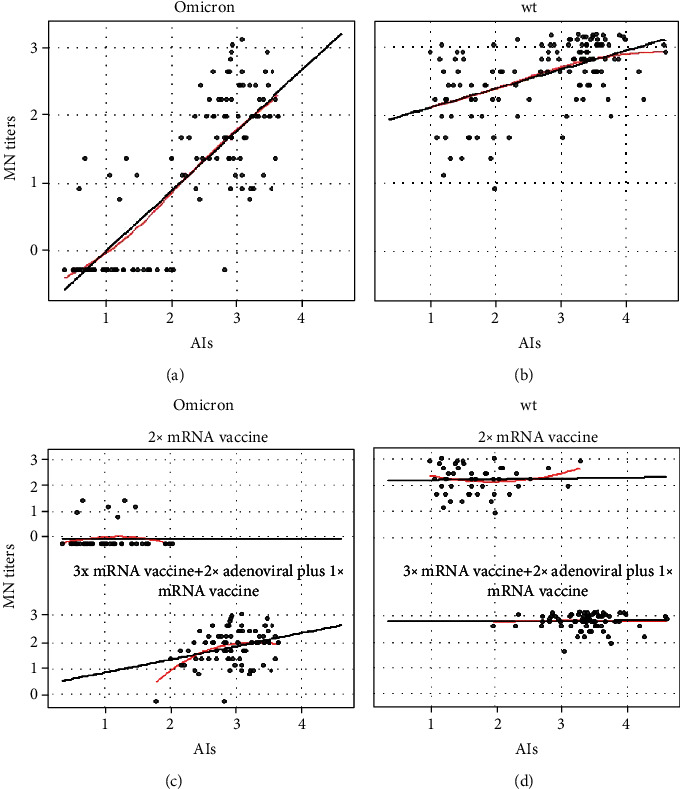
Multiple regression model of antibody avidity and neutralizing antibody titres. Microneutralization (MN) titers are expressed as log2 of the normalized data. Antibody avidity results are expressed as normalized data of avidity index (AI). Regression of MN titers on AI with the virus strain as the dummy variable, Omicron variant (a), and wt virus (b) proved significant for both strains. The number of vaccine doses was included in the regression analysis as a variable: 2 vaccine doses in the top panels, 3 doses in the bottom panels of the Omicron variant (c) and wt virus (d).

A significant association between AIs and neutralizing antibody titers was found (Figures 3(a) and 3(b)) for both the wt virus (slope = 0.28, *p* < 0.0001, *r* = 0.53, and *N* = 124) and Omicron variant (slope = 0.88, *p* < 0.0001, *r* = 0.81, and *N* = 125), without considering the number of vaccine doses received. However, when the number of vaccine doses was included in the model (Figures 3(c) and 3(d)), a significant association between neutralizing antibody titers and AIs (slope = 0.51, *p* = 0.007, *r* = 0.31, and *N* = 77) for the Omicron variant was observed in subjects who had received 3 doses of vaccine. Regarding all the other combinations of virus strain and number of vaccine doses received, the MN results were independent from the AIs.

## 4. Discussion

In this study, we evaluated antibody avidity against the RBD of the wt virus and the Omicron BA.1 variant in serum samples collected from different cohorts of subjects: SARS-CoV-2 infected patients hospitalized during the first pandemic wave in 2020 and subjects who had undergone a course of homologous and/or heterologous vaccination. Vaccinated subjects comprised those who had received 2 doses of mRNA vaccine, those who had received 3 doses of mRNA vaccine, and those who had undergone a primary vaccination cycle with 2 doses of an adenovirus-based vaccine followed by a booster dose of mRNA vaccine.

In patients, the immune response was characterized by an initial increase in both RBD-binding IgG and neutralizing antibodies, followed by a decline. Similarly, the AIs of IgG directed toward the RBD of the wt virus increased over time, but remained somewhat low in the majority of patients. Indeed, only 19.1% of patients showed high AIs during their entire hospital stay. As already observed by previous studies [25–27], after SARS-CoV-2 infection, an initial increase in AIs is followed by a decrease, probably due to incomplete avidity maturation. Failure of the avidity maturation process is manifested by a decline in IgG titers, including neutralizing antibodies, and is possibly due to the limited exposure of the immune system to the antigen.

We found that repeated vaccination was able to induce higher levels of functional antibodies with higher avidity than those induced by natural infection. Both mRNA and adenovirus-based vaccines were designed to express the full-length SARS-CoV-2 S protein in a prefusion state, in order to induce a sustained humoral response in vaccinated subjects [28–30]. As the mechanism of avidity maturation is based on many cycles of mutation and clonal selection, the prolonged availability of antigens seems to be required for proper and complete avidity maturation [25].

For our comparison between SARS-CoV-2 infected patients and vaccinated cohorts, we selected samples collected from patients on day 6 after hospitalization, since these showed the highest neutralizing antibody titers against the wt virus. Although neutralizing antibody titers were higher in patients than in subjects who had received 2 doses of vaccine, and were similar to those seen in subjects who had received 3 doses, avidity showed the lowest values. These results reflect the arrested maturation process described in subjects who have had SARS-CoV-2 infection [25–27] and suggest that the quality of neutralizing antibodies is also affected by avidity maturation. This is even more evident when sera from patients are tested for the Omicron variant, as a marked reduction in the neutralizing antibody response is accompanied by lower avidity values. In a previous study [31], this reduced neutralizing antibody response was observed when these same samples were tested for the alpha (B.1.1.7), beta (B.1.351), and gamma (P.1) variants and was ascribed to the substantial divergence between the infecting strain (wt) and the variants tested. The reduction in neutralizing activity against Omicron and other VoCs was probably due to low affinity, and therefore low avidity, antibodies, confirming that only high-avidity antibodies are involved in virus neutralization, since they can effectively compete with angiotensin-converting enzyme 2 (ACE2) for binding to the RBD [11, 32].

Although neutralizing antibody titers were lower in subjects who had received 2 doses of mRNA vaccine than in patients, these antibodies displayed higher avidity. This observation is in line with previous reports of a significant increase in neutralization and avidity after the administration of a second vaccine dose [17, 33]. However, this immune response was not retained in the case of the Omicron variant, for which both neutralizing titers and avidity were lower. The ability of the Omicron variant to escape the immune response elicited by two vaccine doses observed in this study is consistent with previous reports [34–36].

We observed a significant increase in IgG titers, avidity, and neutralizing antibodies against the wt virus in vaccinated subjects (after 2 and 3 doses of homologous/heterologous vaccine). This suggests that avidity maturation can be sustained by boosting and increases with the number of doses. Thus, the boosting strategy is able to achieve high levels of avidity, which may protect vaccinated subjects, as already observed in one-dose and two-dose studies [17]. The third dose is able to elicit high neutralizing ability and IgG avidity against the Omicron variant, supporting the recommendation for a supplementary dose in order to maintain protection against emerging variants [37, 38]. Although with lower neutralizing titers than those obtained against the wt virus used for vaccines, it is plausible that the booster dose may induce protection from the Omicron variant by reversing antibody decline, generating increased antibody titers that overcome the reduced neutralization associated with the Omicron variant [37]. The observation that high matured affinity strong correlates with neutralizing antibody titers was also observed for other vaccines, such as Dengue vaccine [39].

In this study, no differences in IgG titers or avidity were found between subjects who had received 3 doses of mRNA vaccine and those who had received 2 doses of adenovirus-based vaccine plus a booster dose of mRNA vaccine. This is in contrast with a report that a heterologous vaccination regimen is more immunogenic than a homologous regimen [40]. To point out, samples from subjects who received 3 doses of mRNA vaccine were collected 21 days after the third dose, while samples from subjects who received 2 doses of adenovirus-based vaccine plus a booster dose of mRNA vaccine were collected 42 days after the booster dose. To our knowledge, there are no evidences on differences with affinity maturation between 21 days and 42 days.

The present study has some limitations. Firstly, as serum samples from patients were collected during the first pandemic wave, they might not be representative of the currently infected population. On the contrary, samples from subjects who received 3 doses of vaccine were collected during the Omicron wave, and since no information on SARS-CoV-2 previous infection was available, infection by Omicron cannot be excluded. Moreover, the number of subjects tested was relatively small, and the timing of post-vaccination blood collection did not perfectly match between subjects who had undergone heterologous vaccination and the other cohorts. However, all the cohorts included in this study represented the situation regarding vaccination in a general population.

The evaluation of avidity is an important tool for monitoring vaccine effectiveness. Vaccination seems to play a major role in proper avidity maturation by prolonging the availability of antigens. As the post-vaccination antibody concentration wanes over time, higher avidity may sustain immunity and maintain the ability to fight viral infection at reduced antibody levels [16]. Overall, repeated vaccinations increase antibody avidity towards the mutated S protein of the Omicron variant, supporting the idea that antibodies with high avidity and high neutralizing potential can increase cross-protection against variants that carry several mutations on the RBD.

## Data Availability

The data that support the findings of this study are available from the corresponding author upon reasonable request.

## Supplementary Materials

Supplementary Materials Figure 1S: The figure shows a multiple regression model of RBD-binding antibody titers and neutralizing antibody titrrs. ELISA and microneutralization (MN) titers are expressed as log2 of the normalized data. Regression of MN titrrs on ELISA titers with the virus strain as the dummy variable, Omicron variant, and wt virus proved significant for both strains. The number of vaccine doses was included in the regression analysis as variable 2 vaccine doses and 3 doses of the Omicron variant and wt virus.

## References

[1] Austin Ramzy V. W., Myers S. L., Goldman R. (2020). Naming the coronavirus disease (COVID-19) and the virus that causes it. *Brazilian Journal of Implantology And Health Sciences*.

[2] World Health Organization (2022). Tracking SARS-CoV-2 variants. https://www.who.int/en/activities/tracking-SARS-CoV-2-variants.

[3] SARS-CoV-2 Variant Classifications and Definitions (2021). Centers for disease control and prevention. https://www.cdc.gov/coronavirus/2019-ncov/variants/variant-classifications.html.

[4] Lupala C. S., Ye Y., Chen H., Su X. D., Liu H. (2022). Mutations on RBD of SARS-CoV-2 omicron variant result in stronger binding to human ACE2 receptor. *Biochemical and Biophysical Research Communications*.

[5] Centers for Disease Control and Prevention (2021). Science brief: omicron (B.1.1.529) variant. https://www.cdc.gov/coronavirus/2019-ncov/science/science-briefs/scientific-brief-omicron-variant.html.

[6] Brief T. A., European Centre for Disease Prevention and Control (2021). Implications of the emergence and spread of the SARS-CoV-2 B.1.1 529 variant of concern (Omicron) for the EU/EEA. https://www.ecdc.europa.eu/en/publications-data/threat-assessment-brief-emergence-sarscov-2-variant-b.

[7] Martínez-Flores D., Zepeda-Cervantes J., Cruz-Reséndiz A., Aguirre-Sampieri S., Sampieri A., Vaca L. (2021). SARS-CoV-2 vaccines based on the spike glycoprotein and implications of new viral variants. *Frontiers in Immunology*.

[8] European Commission (2022). Safe COVID-19 vaccines for Europeans.

[9] Brady A. M., Unger E. R., Panicker G. (2017). Description of a novel multiplex avidity assay for evaluating HPV antibodies. *Journal of Immunological Methods*.

[10] Victora G. D., Nussenzweig M. C. (2012). Germinal centers. *Annual Review of Immunology*.

[11] Bauer G. (2021). The potential significance of high avidity immunoglobulin G (IgG) for protective immunity towards SARS-CoV-2. *International Journal of Infectious Diseases*.

[12] Antia A., Ahmed H., Handel A. (2018). Heterogeneity and longevity of antibody memory to viruses and vaccines. *PLoS Biology*.

[13] Lee Y. C., Kelly D. F., Yu L. M. (2008). Haemophilus influenzae type b vaccine failure in children is associated with inadequate production of high-quality antibody. *Clinical Infectious Diseases*.

[14] Bauer G., Struck F., Schreiner P., Staschik E., Soutschek E., Motz M. (2020). The serological response to SARS corona virus-2 is characterized by frequent incomplete maturation of functional affinity (avidity). *Research Square*.

[15] Struck F., Schreiner P., Staschik E. (2022). Incomplete IgG avidity maturation after seasonal coronavirus infections. *Journal of Medical Virology*.

[16] Bliden K. P., Liu T., Sreedhar D. (2021). Evolution of anti-SARS-CoV-2 IgG antibody and IgG avidity post Pfizer and Moderna mRNA vaccinations. *Circulation*.

[17] Pratesi F., Caruso T., Testa D. (2021). BNT162b2 mRNA SARS-CoV-2 vaccine elicits high avidity and neutralizing antibodies in healthcare workers. *Vaccines (Basel)*.

[18] Marchi S., Viviani S., Remarque E. J. (2021). Characterization of antibody response in asymptomatic and symptomatic SARS-CoV-2 infection. *PLoS One*.

[19] Reed L. J., Muench H. (1938). A simple method of estimating fifty per cent ENDPOINTS12. *American Journal of Epidemiology*.

[20] Mazzini L., Martinuzzi D., Hyseni I. (2021). Comparative analyses of SARS-CoV-2 binding (IgG, IgM, IgA) and neutralizing antibodies from human serum samples. *Journal of Immunological Methods*.

[21] Milani G. P., Dioni L., Favero C. (2020). Serological follow-up of SARS-CoV-2 asymptomatic subjects. *Scientific Reports*.

[22] Manenti A., Tete S. M., Mohn K. G. I. (2017). Comparative analysis of influenza a(H3N2) virus hemagglutinin specific IgG subclass and IgA responses in children and adults after influenza vaccination. *Vaccine*.

[23] Moura A. D., da Costa H. H. M., Correa V. A. (2021). Assessment of avidity related to IgG subclasses in SARS-CoV-2 Brazilian infected patients. *Scientific Reports*.

[24] Manenti A., Molesti E., Maggetti M., Torelli A., Lapini G., Montomoli E. (2021). The theory and practice of the viral dose in neutralization assay: insights on SARS-CoV-2 "doublethink" effect. *Journal of Virological Methods*.

[25] Bauer G., Struck F., Schreiner P., Staschik E., Soutschek E., Motz M. (2021). The challenge of avidity determination in SARS-CoV-2 serology. *Journal of Medical Virology*.

[26] Heireman L., Boelens J., Coorevits L., Verhasselt B., Vandendriessche S., Padalko E. (2022). Different long-term avidity maturation for IgG anti-spike and anti-nucleocapsid SARS-CoV-2 in hospitalized COVID-19 patients. *Acta Clinica Belgica*.

[27] Struck F., Schreiner P., Staschik E. (2021). Vaccination versus infection with SARS-CoV-2: establishment of a high avidity IgG response versus incomplete avidity maturation. *Journal of Medical Virology*.

[28] Heinz F. X., Stiasny K. (2021). Distinguishing features of current COVID-19 vaccines: knowns and unknowns of antigen presentation and modes of action. *NPJ Vaccines*.

[29] Xia X. (2021). Domains and functions of spike protein in Sars-Cov-2 in the context of vaccine design. *Viruses*.

[30] Dai L., Gao G. F. (2021). Viral targets for vaccines against COVID-19. *Nature Reviews. Immunology*.

[31] Trombetta C. M., Marchi S., Viviani S. (2021). Serum neutralizing activity against B.1.1.7, B.1.351, and P.1 SARS-CoV-2 variants of concern in hospitalized COVID-19 patients. *Viruses*.

[32] Manuylov V., Burgasova O., Borisova O. (2022). Avidity of IgG to SARS-CoV-2 RBD as a prognostic factor for the severity of COVID-19 reinfection. *Viruses*.

[33] Hollstein M. M., Münsterkötter L., Schön M. P. (2022). Interdependencies of cellular and humoral immune responses in heterologous and homologous SARS-CoV-2 vaccination. *Allergy*.

[34] Muik A., Lui B. G., Wallisch A. K. (2022). Neutralization of SARS-CoV-2 omicron by BNT162b2 mRNA vaccine-elicited human sera. *Science*.

[35] Garcia-Beltran W. F., St Denis K. J., Hoelzemer A. (2022). mRNA-based COVID-19 vaccine boosters induce neutralizing immunity against SARS-CoV-2 omicron variant. *Cell*.

[36] Lusvarghi S., Pollett S. D., Neerukonda S. N. (2021). SARS-CoV-2 Omicron neutralization by therapeutic antibodies, convalescent sera, and post-mRNA vaccine booster. *bioRxiv*.

[37] Richterman A., Scott J., Cevik M. (2021). Covid-19 vaccines, immunity, and boosters. *BMJ*.

[38] Bar-On Y. M., Goldberg Y., Mandel M. (2021). Protection of BNT162b2 vaccine booster against Covid-19 in Israel. *The New England Journal of Medicine*.

[39] Tsuji I., Dominguez D., Egan M. A., Dean H. J. (2022). Development of a novel assay to assess the avidity of dengue virus-specific antibodies elicited in response to a tetravalent dengue vaccine. *The Journal of Infectious Diseases*.

[40] Liu X., Shaw R. H., Stuart A. S. V. (2021). Safety and immunogenicity of heterologous versus homologous prime-boost schedules with an adenoviral vectored and mRNA COVID-19 vaccine (com-COV): a single-blind, randomised, non-inferiority trial. *Lancet*.

