# Supplementary material for: Antibody Avidity and Neutralizing Response against SARS-CoV-2 Omicron Variant after Infection or Vaccination
Source: J Immunol Res. 2022 Aug 31;2022:4813199. doi: 10.1155/2022/4813199 (PMC9453088; doi:10.1155/2022/4813199)
Supplement: Supplementary Materials — Figure 1S: The figure shows a multiple regression model of RBD-binding antibody titers and neutralizing antibody titrrs. ELISA and microneutralization (MN) titers are expressed as log2 of the normalized data. Regression of MN titrrs on ELISA titers with the virus strain as the dummy variable, Omicron variant, and wt virus proved significant for both strains. The number of vaccine doses was included in the regression analysis as variable 2 vaccine doses and 3 doses of the Omicron variant and wt virus. [file 4813199.f1.pdf]

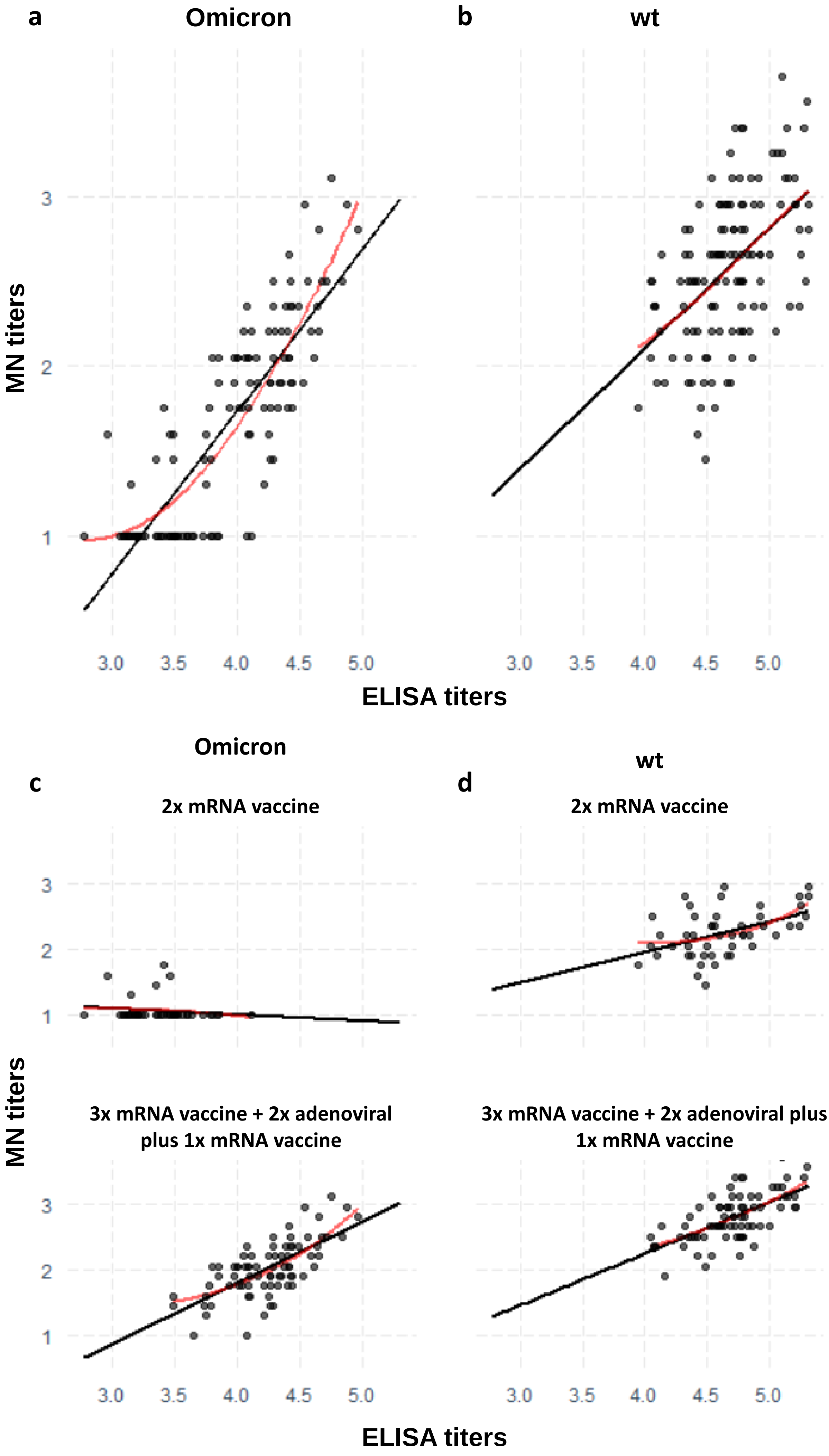

Figure 1 supplementary. Multiple regression model of RBD-binding antibody titres and neutralizing antibody titres. ELISA and microneutraliziation (MN) titres are expressed as log2 of the normalized data. Regression of MN titres on ELISA titres with the virus strain as the dummy variable, Omicron variant (a) and wt virus (b) proved significant for both strains. The number of vaccine doses was included in the regression analysis as a variable 2 vaccine doses in the top panels, 3 doses in the bottom panels of the Omicron variant (c) and wt virus (d).
